# A single genomic region controls primocane fruiting in tetraploid blackberry

**DOI:** 10.1093/genetics/iyag078

**Published:** 2026-03-23

**Authors:** Alexander Silva-Córdoba, Isabella Vaughn, T Mason Chizk, Lacy Nelson, Carmen Johns, Ellen Thompson, Nahla Bassil, Michael Hardigan, John Clark, Tomas Brůna, Marcelo Mollinari, Margaret Worthington

**Affiliations:** Department of Horticulture, University of Arkansas System Division of Agriculture, 316 Plant Science Building, Fayetteville, AR 72701, United States; Department of Horticulture, University of Arkansas System Division of Agriculture, 316 Plant Science Building, Fayetteville, AR 72701, United States; Department of Horticulture, University of Arkansas System Division of Agriculture, 316 Plant Science Building, Fayetteville, AR 72701, United States; Department of Horticulture, University of Arkansas System Division of Agriculture, 316 Plant Science Building, Fayetteville, AR 72701, United States; Department of Horticulture, University of Arkansas System Division of Agriculture, 316 Plant Science Building, Fayetteville, AR 72701, United States; Hortifrut Genetics Ltd., PO Box 1540, Freedom, CA 95019, United States; United States Department of Agriculture, Agriculture Research Service, National Clonal Germplasm Repository, 33447 Peoria Road, Corvallis, OR 97333, United States; United States Department of Agriculture, Agriculture Research Service, Horticultural Crops Production and Genetic Improvement Research Unit, 3420 NW Orchard Avenue, Corvallis, OR 97330, United States; Department of Horticulture, University of Arkansas System Division of Agriculture, 316 Plant Science Building, Fayetteville, AR 72701, United States; U.S. Department of Energy, Joint Genome Institute, Lawrence Berkeley National Laboratory, Berkeley, CA 94720, United States; Department of Horticultural Science, North Carolina State University, Kilgore Hall, 2721 Founders Dr, Raleigh, NC 27607, United States; Department of Horticulture, University of Arkansas System Division of Agriculture, 316 Plant Science Building, Fayetteville, AR 72701, United States

**Keywords:** annual flowering, GWAS, *Rubus*, Rosaceae, polyploidy, linkage mapping, KASP marker

## Abstract

The fresh-market blackberry (*Rubus* subgenus *Rubus*) industry has expanded dramatically in the past 2 decades, driven in part by improved cultivars. Introgression of the primocane-fruiting (PF; annual flowering) trait into elite germplasm has enabled dual cropping in a single year, season extension, and cultivation in tropical and subtropical regions. Despite its economic performance, the genetic basis of PF is not well understood. It has been proposed that the PF trait is controlled by a major recessive locus, but its genomic location is unclear. Here, a genome-wide association study (GWAS) of 365 tetraploid blackberry genotypes identified a single genomic region on chromosome Ra03 (∼33 Mb) strongly associated with PF. Genetic linkage analysis in a biparental population confirmed that the same interval (32–35 Mb) was linked to the PF phenotype. Ten putative candidate genes were identified in this region. Allele mining using whole-genome resequencing of 17 genotypes highlighted 2 high-priority candidates: a CCCH-type zinc finger gene and an ubiquitin-specific protease gene. Use of an improved *Rubus argutus* “Hillquist” genome annotation (v1.2) enabled refined variant interpretation, including identification of regulatory 3′ UTR polymorphisms in the zinc finger homolog. Two diagnostic KASP markers (*PF1* and *PF2*), designed from the most significant GWAS SNPs, predicted the PF phenotype with over 96% accuracy in a validation panel of 494 tetraploid blackberries from multiple breeding programs. Together, these results provide the first high-resolution mapping of the PF locus in blackberry, identify candidate genes for flowering regulation in *Rubus*, and deliver diagnostic markers that can be immediately deployed in breeding programs.

## Introduction

**Fig. 1.**
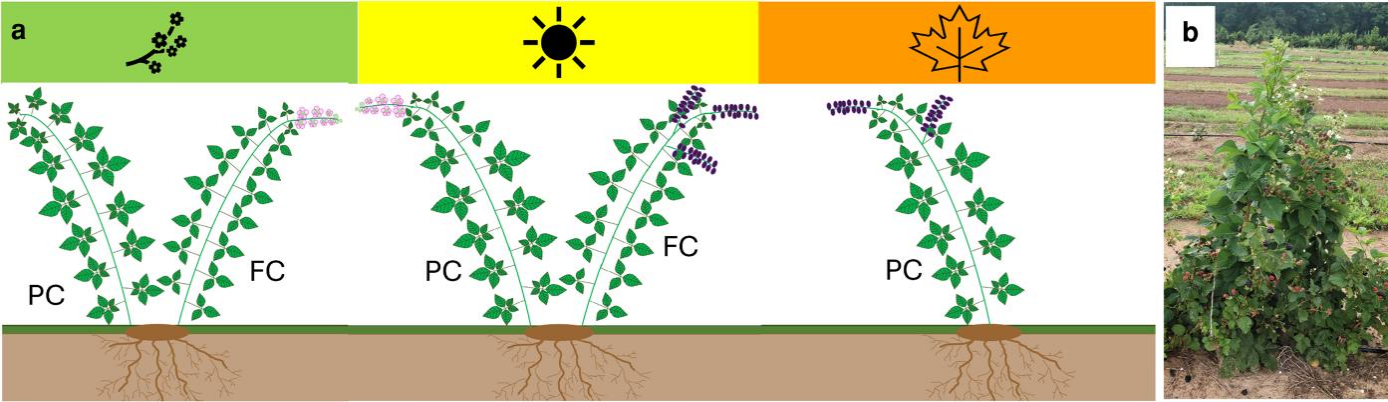
Flowering and fruiting patterns in primocane-fruiting (PF) blackberry genotypes. (a) Diagram illustrating seasonal development of canes in PF cultivars under double-cropping management, shown as a sequence from spring to fall (left to right). Primocanes (PC) are vegetative in spring, flower on a portion of the cane in summer, and produce fruit in late summer or fall. Portions of the primocane that remain vegetative initiate floral buds in autumn, overwinter, and flower the following spring when that cane functions as a floricane (FC). The senesced portion of the cane that fruited as a primocane does not contribute to the subsequent floricane crop. (b) PF genotype S442 showing flowers and unripe fruit on primocanes in the upper canopy with ripe floricane fruit below.

Blackberry (*Rubus* subgenus *Rubus*) is a specialty crop of increasing economic significance due to rising consumption, expanded marketing initiatives, and advancements in cultivar development (Clark and Finn 2014). Blackberries are herbaceous or semi-woody plants with a perennial crown root system and biennial canes. The first-year canes (primocanes), are typically vegetative, while the second-year canes (floricanes), produce flowers and fruits after a period of dormancy requiring varying degrees of chilling hours (often during winter in temperate environments). Genotypes that produce fruit exclusively on floricanes are known as floricane-fruiting (FF) or biennial-flowering cultivars. In contrast, primocane-fruiting (PF) or annual-flowering cultivars flower and fruit on new canes produced each year (Clark 2008) (Fig. 1). Primocane-fruiting is a trait also present in other *Rubus* crops and has been critical for the expansion of red raspberry (*R. ideaus*) production (Clark et al. 2007). In blackberry, the PF trait was initially found in a wild *R. argutus* accession named “Hillquist” (PI 553951). This diploid blackberry accession was used as the initial donor parent to develop tetraploid PF cultivars at the Fruit Breeding Program of the University of Arkansas System Division of Agriculture (UADA), which released the first PF cultivars (Prime-Jan® and Prime-Jim®) in 2004 (Clark et al. 2005). PF cultivars have some advantages over traditional floricane-fruiting cultivars, such as extended production season in the autumn or a double-cropping system (a first harvest season on floricanes in early summer and a second season on primocanes in late summer and fall), scheduled production based on primocane management, avoidance of winter injury in colder areas, and expanded production in regions where chilling requirements are not entirely fulfilled (Clark and Finn 2014).

Although PF blackberry cultivars are widely adopted in the fresh-market raspberry and blackberry industries, the molecular mechanism and genes controlling this trait have not been well elucidated in either species. Several studies have been conducted to understand the inheritance of primocane fruiting in tetraploid blackberries. Lopez-Medina et al. (2000) evaluated PF segregation ratios in a full diallel crossing scheme and proposed that PF is controlled by a single recessive gene. In addition, Castro et al. (2013) constructed the first genetic map of tetraploid blackberry from a cross between Prime-Jim® (PF cultivar) and “Arapaho” (FF cultivar) using 119 simple sequence repeat (SSR) markers. They identified a locus linked with the PF trait in this segregating population on linkage group (LG) 7 between the markers RH_MEa0006aC04-175 and RH_MEa0007aG06-152. However, it was later shown that most of the markers on LG7, including the flanking markers to the PF locus, aligned on chromosome 2 in the black raspberry (*R. occidentalis*) genome (VanBuren et al. 2016) and the blackberry “Hillquist” reference genome (Brůna et al. 2023). The genetic control of the PF trait has been investigated in red raspberry as well; Jibran et al. (2019) reported 2 quantitative trait loci associated with PF in red raspberry, *RiAF3* and *RiAF4* on linkage groups 3 and 4, respectively, using a biparental population from the cross between the PF accession NC493 (*R. parvifolius* × *R. idaeus “*Cherokee’) and the FF *R. idaeus* genotype “Chilliwack”. This finding suggested that PF in raspberries is a complex trait controlled by more than 1 locus, in contrast to previous findings in tetraploid blackberries.

The development of genomic resources for blackberries is critical for the elucidation of the genetic control of important agronomic traits such as PF. Two reference quality assemblies of blackberry have recently become available. The first, from the diploid donor of the PF trait (*R. argutus* cv. “Hillquist”) was assembled at the chromosome scale using Pacific Biosciences long reads, with 38,503 predicted protein-coding genes, 72% of which were functionally annotated (Brůna et al. 2023). The second, from the PF thornless tetraploid blackberry selection BL1, was assembled and haplotype-phased at the chromosome scale using Oxford Nanopore long reads and Hi-C scaffolding, yielded 27 pseudochromosomes and 87,968 predicted protein-coding genes, 82% with functional annotation (Paudel et al. 2025). Together, these genomic resources provide a robust foundation for the identification of loci and candidate genes controlling PF and other complex traits in blackberry.

Next-generation sequencing (NGS) technologies have enabled the generation of large sets of markers with sufficient read depth to accurately estimate allele dosages in polyploid crops using software such as the R package Updog (Gerard et al. 2018), which accounts for variable read depth, sequencing errors, allele bias, and overdispersion. Recently, Capture-Seq genotyping (RAPiD Genomics) was performed using 35,054 biotinylated probes distributed across the “Hillquist” reference genome to identify 124,564 biallelic SNPs across a diverse panel of 502 blackberry genotypes (Chizk et al. 2023). This tetraploid blackberry panel and set of SNPs have been used to identify loci controlling fruit firmness, red drupelet reversion, soluble solid content, pH, acidity, and the prickle-free trait in tetraploid blackberry (Chizk et al. 2023; Godwin et al. 2025; Johns et al. 2025).

Genome-wide association studies (GWAS) and linkage mapping have been extensively used across various species to identify loci associated with diverse traits. The development of the GWASpoly software (Rosyara et al. 2016), designed specifically for autopolyploids to model different types of polyploid gene action, including additive and simplex dominant, has enabled the identification of genomic regions in crops such as blueberry (Ferrão et al. 2018), potato (Sharma et al. 2018), sweet potato (Wilson et al. 2021), sugarcane (Saavedra-Díaz et al. 2024), and blackberry (Chizk et al. 2023; Godwin et al. 2025; Johns et al. 2025). Similarly, the development of the R package MAPpoly (Mollinari and Garcia 2019) has facilitated the efficient construction of linkage maps using thousands of SNP markers in autopolyploid species, such as in blueberry (Cappai et al. 2020), sweetpotato (Mollinari et al. 2020; Oloka et al. 2021), potato (da Silva et al. 2021a, 2021b), and rose (Lau et al. 2022).

In this study, a panel of 365 diverse tetraploid blackberry genotypes and a segregating biparental population were used to identify genomic regions linked to the PF trait through GWAS and linkage mapping. Based on these analyses, diagnostic Kompetitive Allele-Specific PCR (KASP) markers were developed and validated in a diverse blackberry panel. The most predictive KASP markers have practical applications in breeding programs for marker-assisted selection. Furthermore, identification of candidate genes and allele mining using whole-genome sequence data and an improved annotation of *R. argutus* cv. “Hillquist” enhances the understanding of the genetic control underlying the PF trait.

## Materials and methods

### Plant material

A diverse panel of 365 tetraploid blackberry cultivars and selections from the UADA breeding program were classified as PF or FF based on the presence or absence of flowers on first-year canes. Phenotyping was conducted at the plot level rather than on individual plants, as clonally replicated plants were grown at close spacing and primocanes frequently intermingled. All canes within each plot were evaluated. Each cultivar and selection was maintained in a 6 m plot with an average of 10 plants per plot at the UADA Fruit Research Station (FRS), Clarksville, AR, located at 35°31′5″ N and long. 93°24′12″ W.

Plots were evaluated weekly from late May to July between 2019 and 2023. Plots were evaluated between 1 and 5 years depending on breeding decisions and plot longevity. Genotypes were classified as PF if at least 20% of primocanes within the plot produced flowers in at least 1 year. This qualitative threshold was chosen to capture genetic capacity for annual flowering while minimizing misclassification due to heat suppression of flowering in weak PF genotypes. In ambiguous cases, canes were examined at their point of origin to distinguish true primocanes arising from the crown from late basal shoots originating on floricanes. Leaf morphology was also used to distinguish true primocanes from basal shoots; primocanes have 5 leaflets during vegetative growth and transition to 3 leaflets a few nodes before flowering, while floricanes have 3 leaflets along the entire cane.

As a complementary strategy, genetic linkage analysis was conducted to identify genomic regions associated with the PF trait. A blackberry biparental mapping population was generated in 2019 by crossing the FF selection “S16” and the PF selection “S242”. The resulting progeny were planted at 0.6 m spacing at FRS in Spring 2020. Individual seedlings were scored for the presence or absence of flowers on primocanes during June and July of 2022 and 2023. Scoring at the single-plant level was feasible in this population due to the strong and consistent primocane flowering phenotype of the PF parent (S242) and its progeny.

### Genotyping and SNP calling

The tetraploid blackberry diversity panel was previously genotyped through the Capture-Seq technology by RAPiD Genomics (Gainesville, FL), as described in Chizk et al. (2023). Briefly, DNA from young leaf tissue of each genotype was extracted using a cetyltrimethylammonium bromide (CTAB) protocol modified from Porebski et al. (1997). A Qubit dsDNA assay kit (Invitrogen, Carlsbad, CA) was used to quantify DNA concentration. A set of 35,054 custom biotinylated 120-mer probes distributed along the “Hillquist” (*R. argutus*) reference genome (Brůna et al. 2023) were used to sequence each genotype. DNA libraries were sequenced using an Illumina HiSeq2000 instrument with an average coverage of 150×. Illumina paired-end reads were cleaned, trimmed, and aligned to the reference genome using MOSAIK v2.2.30 (Lee et al. 2014), and diploid variants were detected using Freebayes v1.3.1 (Garrison and Marth 2012). VCFtools (Danecek et al. 2011) was used to select biallelic SNPs with a minor allele frequency ≥0.01. Tetraploid allele dosage was estimated using the multidog function from Updog v2.0.3 (Gerard et al. 2018) based on the filtered VCF file.

The biparental population was genotyped using Allegro targeted genotyping (Tecan Genomics, Redwood City, CA, USA). DNA from young leaf tissue of parents and 165 seedling progenies was extracted using a CTAB protocol modified from Porebski et al. (1997) as previously described. A custom set of 46,485 probes was developed to target 25,000 sites across the *R. argutus “*Hillquist” reference genome. Of these targets, 23,076 were fully covered and 433 were partially covered. Sequencing libraries were prepared following the Allegro protocol and sequenced using an Illumina NextSeq 2000 to generate 100-bp single-end reads. The sequencing reads were cleaned and trimmed using Trimmomatic v0.39 (Bolger et al. 2014) and aligned to the “Hillquist” reference genome using Burrows-Wheeler Aligner (bwa)-mem2 v0.7.17 software (Li and Durbin 2009). Genetic variants were called using GATK v4.2.6.1 HaplotypeCaller with the tetraploid option (-ploidy 4), followed by joint genotyping for the whole population with the GenotypeGVCFs tool in GATK (Poplin et al. 2018). SNPs with low confidence were filtered using the following parameters: Phred-scaled quality score below 30 (QUAL < 30), normalized quality score below 2 (QUAL/DP < 2.0), mapping quality below 40 (MQ < 40.0), or depth of coverage less than 50 for each sample (FMT/DP < 50).

### Genome-wide association analysis

GWAS was conducted using the GWASpoly v2.30 (Rosyara et al. 2016) R package under the K model to control population structure. The K matrix was constructed using the leave-one-chromosome-out (LOCO) method, in which a covariance matrix is calculated for each chromosome based on the markers from all other chromosomes. Based on previous research demonstrating that PF is controlled by a single recessive locus in blackberry (Lopez-Medina et al. 2000; Castro et al. 2013), the complete dominance (1-dom) model of gene action was tested. A maximum genotype frequency of 0.98 was set. The “M.eff” method was used to establish −log10(*P*) significance thresholds of 4.93, and 5.5 for the complete alternative allele dominance and complete reference allele dominance models, respectively, to control the genome-wide false discovery rate. QQ-plots of observed vs expected *P*-values under the null hypothesis were constructed to assess potential inflation of the test statistics.

### Linkage mapping for the primocane-fruiting locus

A genetic linkage map for the biparental population was constructed using the R package MAPpoly2 (https://github.com/mmollina/mappoly2; Mollinari and Garcia 2019). SNPs and progenies with more than 10% missing data were excluded. The chi-square (χ^2^) test was used to assess segregation distortion, assuming only random chromosome bivalent pairing (no double reduction). Redundant markers were automatically removed before computing the pairwise recombination fraction between all the selected markers using a 2-point analysis. The recombination fraction between the PF phenotype and each molecular marker was also calculated. Markers were grouped using 2 sources of information: the chromosome information from the physical map of the *R. argutus* cv. “Hillquist” genome, and the recombination fractions between markers using the Unweighted Pair Group Method with Arithmetic Mean (UPGMA) clustering method. The function “make_sequence” of MAPpoly2 was used to combine the UPGMA-derived groups and the chromosome assignments to ensure the most consistent and supported grouping.

The multidimensional scaling (MDS) method, based on pairwise recombination fractions, was used to determine the order of the markers within each group. The genome-based ordering was included in the analysis to refine the order of markers. Linkage phase was estimated using the function “pairwise_phasing” which utilizes pairwise recombination information to identify a set of linkage phase configurations for each parent. Individual genetic maps were constructed for each parent using a multilocus approach in which the entire chromosome structure is considered; misplaced markers or incorrect linkage phases can be detected in this approach as an inflation of the expected map length. Individual maps were integrated using a Hidden Markov Model (HMM) model, considering a global genotyping error of 5%. MAPpoly2 also calculated homologous pairing probabilities, allowing for detection of preferential pairing during meiosis.

### Structural annotation of *R. argutus* cv. “Hillquist” v.1.2

An updated gene annotation was generated for the *R. argutus “*Hillquist” genome assembly in collaboration with the Joint Genome Institute (JGI). While no new genomic or transcriptomic sequence data were generated, existing Iso-Seq and RNA-Seq datasets (Brůna et al. 2023) were reanalyzed in the pipeline, resulting in substantially improved gene models, including more complete untranslated regions (UTRs), refined exon and intron structures, and updated functional annotations. Transcript assemblies were derived from ∼136 M pairs of 2 × 100 stranded paired-end Illumina RNA-seq reads. Reads were aligned to the genome using GSNAP within the PERTRAN framework, which constructs splice alignment graphs after alignment validation, realignment, and correction (Wu and Nacu 2010). Approximately 2.5 M PacBio Iso-Seq nonchimeric CCSs were processed with a genome-guided correction pipeline, aligned with GMAP (Wu and Nacu 2010) to correct small InDels at splice junctions, and clustered based on intron structure or ≥95% overlap for single-exon transcripts, yielding roughly 240,000 full-length transcripts. mina- and PacBio-derived assemblies were then integrated and refined with PASA (Haas et al. 2003), producing 152,786 transcript models prior to filtering.

The genome was soft-masked using RepeatMasker (Smit et al. 2013) with a species-specific repeat library. This library combined de novo repeats predicted by RepeatModeler2 (Flynn et al. 2020) from the *R. argutus* and *R. ulmifolius* genomes, together with common Viridiplantae and Embryophyta repeats from RepBase (Bao et al. 2015) and Dfam (Hubley et al. 2016). Putative gene loci were inferred from transcript alignments and protein homology. Proteins from 24 diverse plant species (*Arabidopsis thaliana*, *Beta vulgaris*, *Cannabis sativa*, *Carya illinoinensis*, *Cucumis sativus*, *Fragaria vesca*, *Glycine max*, *Gossypium raimondii*, *Liriodendron tulipifera*, *Malus domestica*, *Medicago truncatula*, *Mimulus guttatus*, *Morus notabilis*, *Oryza sativa*, *Populus trichocarpa*, *Potentilla anserina*, *Prunus persica*, *Rosa chinensis*, *Solanum lycopersicum*, *Sorghum bicolor*, *Vitis vinifera*, and *Ziziphus jujuba*), along with Swiss-Prot eukaryotic proteins (release 2022_04), were aligned to the repeat-masked genome with EXONERATE (Slater and Birney 2005), allowing extensions up to 2 kb beyond predicted gene boundaries when nonoverlapping with other loci.

Gene models were predicted using multiple approaches, including FGENESH+ and FGENESH_EST (Salamov and Solovyev 2000), EXONERATE, AUGUSTUS (Stanke et al. 2006), and PASA-derived homology-constrained ORFs. AUGUSTUS was trained on high-confidence PASA-derived ORFs and intron hints from short read alignments. At each locus, the highest scoring prediction was selected based on transcript and protein support while avoiding predictions overlapping with repeats. PASA further refined models by adding UTRs, alternative transcripts, and correcting splice junctions.

Gene model proteins were evaluated for homology against the reference proteomes to calculate Cscores (the ratio of a protein's BLASTP score to that of its mutual best-hit) and protein coverage (percentage aligned to best homolog). Transcripts were retained if they were supported by transcripts derived from Iso-Seq and RNA-Seq or had Cscore and coverage ≥0.5. Stricter thresholds (Cscore ≥0.9 and coverage ≥70%) were applied for models with >20% CDS overlap with repetitive elements. Proteins were also annotated for domains using Pfam (Mistry et al. 2021), and models with >30% overlap with TE-related domains or lacking transcript/homology support were removed. Additional manual curation excluded incomplete or weakly supported gene models, short single-exon models (CDS <300 bp) without recognizable domains or expression evidence, and repetitive models lacking strong homology support.

### Functional annotation of *R. argutus* cv. Hillquist v.1.2

All predicted peptide sequences were functionally annotated using a computational pipeline. InterProScan 5 (Jones et al. 2014) was used to identify protein domains and sequence features, which were subsequently used to assign Gene Ontology (GO) terms. Enzyme functions were predicted with E2P2 (Chae et al. 2014; Schläpfer et al. 2017) to assign EC numbers, and PathoLogic (Karp et al. 2011) was employed to map proteins to metabolic pathways. Eukaryotic Orthologous Groups (KOG) classifications were assigned using a modified mutual best-hit algorithm. Finally, protein domain annotations were integrated to generate putative gene functional assignments, including the multiplicity of each function across the proteome.

### Whole-genome sequencing of PF and FF genotypes

A set of 17 diverse tetraploid blackberry genotypes was selected for whole-genome resequencing with the aim of identifying functional polymorphisms (SNPs and small InDels) responsible for the PF trait. Young leaf tissue of the PF genotypes Black Magic®, Prime-Ark® “45”, Prime-Ark® “Freedom”, “S213”, “S214”, and “S242”, and the- FF genotypes “Apache”, “Kiowa”, “Osage”, “Ouachita”, “Natchez, “Navaho”, “Tupy”, “S9”, “S17”, “S28” and “S334” was collected on ice and stored at −80 °C until DNA isolation. Sequencing and raw data analysis were previously described by Johns et al. (2025). Briefly, 150 bp paired-end reads were cleaned and trimmed with Trimmomatic (Bolger et al. 2014) and aligned to the *R. argutus* cv. “Hillquist” v.1.2 reference genome using bwa-mem2 software (Vasimuddin et al. 2019). Genetic variants were called using GATK using the option ploidy (-ploidy 4) of the “HaplotypeCaller” function for tetraploid calling. Finally, functional annotation of genetic variants was performed using NGSEP (Duitama et al. 2014), based on the GFF3 file containing gene functional annotations for the “Hillquist” v.1.2 genome.

### Identification of primocane-fruiting candidate genes

To identify candidate genes responsible for the PF trait, linkage disequilibrium (LD) was estimated between SNPs on chromosome Ra03 using the ldsep R package (Gerard 2021). Based on the observed slow rate of LD decay, a genomic region surrounding the most significant PF-associated SNP was selected for further comparison between PF and FF genotypes. Genes annotated in the *R. argutus* cv. “Hillquist” v1.2 genome within this region with a gene function associated with flowering were considered candidate genes. Candidate genes were further evaluated using whole-genome sequencing data to identify SNPs and InDels predicted to alter the encoded protein between PF and FF genotypes.

### KASP marker development and validation

Whole-genome sequence data were used to identify polymorphisms within the genomic region associated with the PF trait in the GWAS and genetic linkage analysis. These polymorphisms were then used to develop diagnostic Kompetitive Allele-Specific PCR (KASP) markers for predicting the PF trait. Because the WGS data were aligned to the “Hillquist” reference genome, the original donor of the PF trait, and it has been reported that a single recessive allele controls the PF trait (Lopez-Medina et al. 2000), SNPs were selected based on genotype patterns consistent with this inheritance. In the 6 PF genotypes, selected SNPs had to be homozygous for the reference allele (AAAA, where “A” denotes the reference allele), whereas in the 11 FF genotypes, the selected SNPs were required to be heterozygous or homozygous for the alternative allele (AAAB, AABB, ABBB, or BBBB, where “B” represents the alternative allele). Twelve SNPs located within 1 Mb of the most significant GWAS peak were selected as targets for KASP assay design by LGC Genomics (Beverly, MA, USA). Each KASP assay contains 2 allele-specific forward primers, each with a unique tail sequence serving as a binding site for oligonucleotides labeled with fluorescent dyes FAM or HEX, and 1 common reverse primer.

To evaluate the accuracy of the KASP markers to predict the PF trait, a set of 494 genotypes with diverse genetic backgrounds from the UADA program (including 165 seedlings from the biparental population 1937 and 201 cultivars and selections), the United States Department of Agriculture—Horticultural Crops Production and Genetic Improvement Research Unit (USDA-ARS-HCPGIRU, *n* = 76), Hortifrut Genetics (HFG, *n* = 39), and the United States Department of Agriculture—National Clonal Germplasm Repository (USDA-ARS-NCGR, *n* = 13), was genotyped at LGC Genomics (Beverly, MA, USA). A total of 167 out of the 367 genotypes included in the GWAS panel were also represented in the validation panel, comprising 155 cultivars and selections from UADA, 2 cultivars from USDA-ARS HCPGIR, and 10 accessions from USDA-ARS-NCGR. KASP genotyping was performed by LGC Biosearch Technologies as described in Johns et al. (2025). To determine allele dosage, the fluorescence values measured for the FAM and HEX dyes for each KASP marker were analyzed using SNPviewer software (Biosearch Technologies). Final cluster plots were generated using the ggplot R package (Wickham 2016).

## Results

### GWAS reveals a major locus on chromosome Ra03 controlling the PF trait

**Fig. 2.**
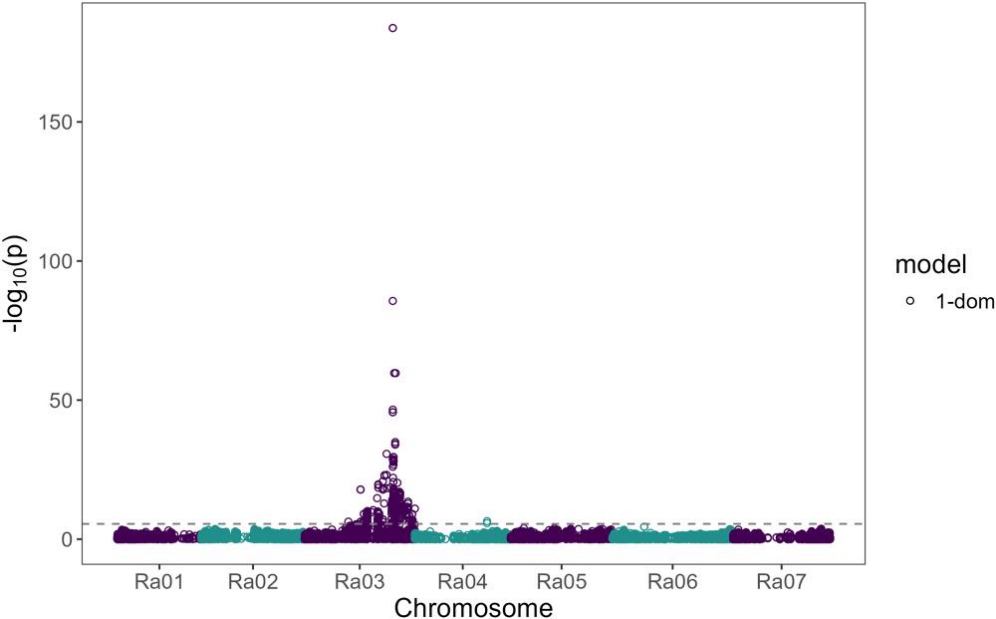
Manhattan plot for the association analysis between primocane-fruiting trait and SNPs under the simplex-dominant (1-dom) model. The dashed line represents the −log10(*P*) threshold using the “M.eff” method implemented in GWASpoly.

A set of 137 PF and 228 FF blackberry genotypes were used in the genome-wide association study (Supplementary Table 1). Of the 124,564 SNPs initially identified in a larger panel of 495 blackberry genotypes, 81,064 biallelic SNPs distributed across the 7 blackberry chromosomes were retained for association analysis in 365 genotypes. The final set of markers was obtained after filtering those with a maximum genotype frequency higher than 1–5/*N*, where *N* represents the population size, as recommended for heterozygous panels by Rosyara et al. (2016). Chromosome-specific QQ-plots revealed an upward curve only on chromosome Ra03, indicating no technical biases or population structure affecting the association (Supplementary Fig. 1). Using the “M.eff” method to control the genome-wide false positive rate, 419 significant SNPs associated with the PF trait were detected within a genomic region located on chromosome Ra03 between 21,158,993 and 41,668,169 bp (Supplementary Table 2). The most strongly associated SNPs were located at 33,338,602 bp and 33,338,650 bp, each with a −log_10_(*P*) of 183.8 under the simplex dominant (1-dom-ref) model (Fig. 2). Both variants are located within an intron of the gene Ruarg.3G335600, which is homologous to the gene AT2G28470 that encodes the enzyme Beta-galactosidase 8 (BGAL8) in *A. thaliana*. For both SNPs, 133 out of 137 PF blackberry genotypes (97%) were homozygous for the reference allele (the allele associated with the presence of the PF trait). In contrast, all FF genotypes were either homozygous for the alternative allele or heterozygous.

### Genetic linkage mapping supports placement of PF locus on chromosome Ra03

The first integrated SNP-based linkage map in blackberry was constructed to identify loci associated with the PF trait. A total of 165 seedlings derived from the cross between the FF selection “S16” and the PF selection “S242” were evaluated for the presence of flowering primocanes during 2 consecutive years and submitted to Allegro targeted genotyping. Ninety progenies were recorded as PF, 72 as FF, and 3 were undetermined. Thirty-six progenies with a low number of Allegro targeted genotyping reads were excluded from the linkage analysis. After filtering out SNPs with low quality, 9,736 SNP markers were initially selected to construct the genetic linkage map (Supplementary Table 3). The most strongly associated SNP markers identified in the GWAS (Ra03:33,338,650 and Ra03:33,338,602) were also evaluated in the 1937 biparental population (see Development and Validation of KASP Markers section). However, Ra03:33,338,650 was excluded from linkage mapping due to missing data in 13 out of the 113 progenies (more than 10%). A Principal Component Analysis (PCA) was performed to identify potential self-cross individuals or contaminants in the biparental population. As a result, 16 individuals were identified as potential nontrue F_1_ samples and were excluded from the analysis (Supplementary Fig. 2). Therefore, the linkage map was constructed using 113 F_1_ seedlings, of which 62 were recorded as PF, 50 were recorded as FF, and 1 was undetermined.

Because both “S16” and “S242” were included in the GWAS panel, allele dosages for the most significant markers linked to the PF trait could be estimated for both parents. The FF female parent, “S16”, carries 3 copies of the reference allele associated with the PF trait, as indicated by genotypes at markers Ra03:33,338,650_G/A (GGGA) and Ra03:33,338,602_T/C (TTTC). The PF male parent, “S242”, was homozygous for the PF allele at both loci, with genotypes GGGG and TTTT, respectively (Supplementary Table 1). Assuming segregation from a triplex (3 PF alleles) × quadriplex (4 PF alleles) cross, the observed PF:FF segregation ratio of the offspring fit the expectation for a tetrasomic inheritance model for a trait controlled by a single recessive allele, under both random chromosome assortment (expected ratio 1:1; χ^2^ = 1.286; *P* = 0.257) and random chromatid assortment (expected ratio 15:13; χ^2^ = 0.144; *P* = 0.705).

**Fig. 3.**
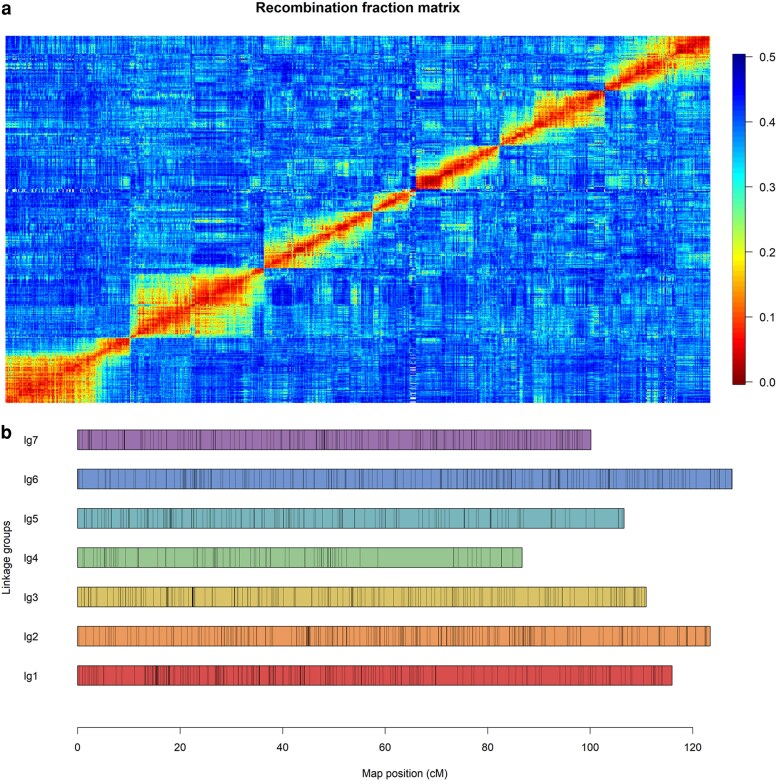
Linkage map construction for the blackberry biparental population 1937 derived from the cross between the FF genotype “S16” and the PF genotype “S242”. a) Recombination fraction matrix showing 7 submatrices corresponding to each of the 7 linkage groups (basic chromosome number in blackberry). b) Integrated linkage groups (LGs) constructed by combining the individual genetic maps from both parents.

After filtering markers with >10% missing data, monomorphic loci, redundant markers, and those showing significant segregation distortion (χ^2^ test, *P* < 0.05), a final set of 3,882 SNPs was retained for linkage mapping using MAPpoly2. Pairwise recombination fractions were computed, and the resulting matrix exhibited the expected 7 block-diagonal clusters corresponding to the 7 blackberry linkage groups (LGs) (Fig. 3a). The genetic map was assembled assuming a 5% global error rate and using the “Hillquist” reference genome to guide marker order (Mollinari and Garcia 2019). The final map spanned 769.4 cM and included 3,197 nonredundant SNPs, averaging 4 markers per cM across LGs ranging from 86.5 cM (LG4) to 122.2 cM (LG2) (Fig. 3b, Table 1). Marker density was highest in LG2 (618 SNPs; 5.06 markers/cM) and lowest in LG4 (208 SNPs; 2.41 markers/cM), with the largest gaps observed in LG4 (14.7 cM) and LG5 (5.3 cM). The map was highly collinear with the “Hillquist” physical map, showing no apparent inversions or translocations (Supplementary Fig. 3). Probability profiles of all meiotic pairing configurations indicated no evidence of preferential pairing in either parent (Supplementary Fig. 4).

**Table 1.** Genetic map summary of the tetraploid blackberry biparental population 1937, derived from a cross between the floricane-fruiting (FF) genotype “S16” and primocane-fruiting (PF) genotype “S242”.

LG	Map length (cM)	Markers/cM	Simplex _P1	Simplex_P2	Double-simplex	Multiplex	Total	Max gap
1	115.9	4.94	234	40	95	204	573	4.3
2	122.2	5.06	26	26	268	298	618	3.7
3	110.5	4.24	53	86	103	226	468	3.3
4	86.5	2.41	44	52	39	73	208	14.7
5	106.0	3.52	61	10	46	256	373	5.3
6	128.0	3.91	66	41	165	228	500	3.1
7	110.3	4.56	46	72	144	195	457	3.2
Total	769.4	4.00	530	327	860	1480	3197	14.7

**Fig. 4.**
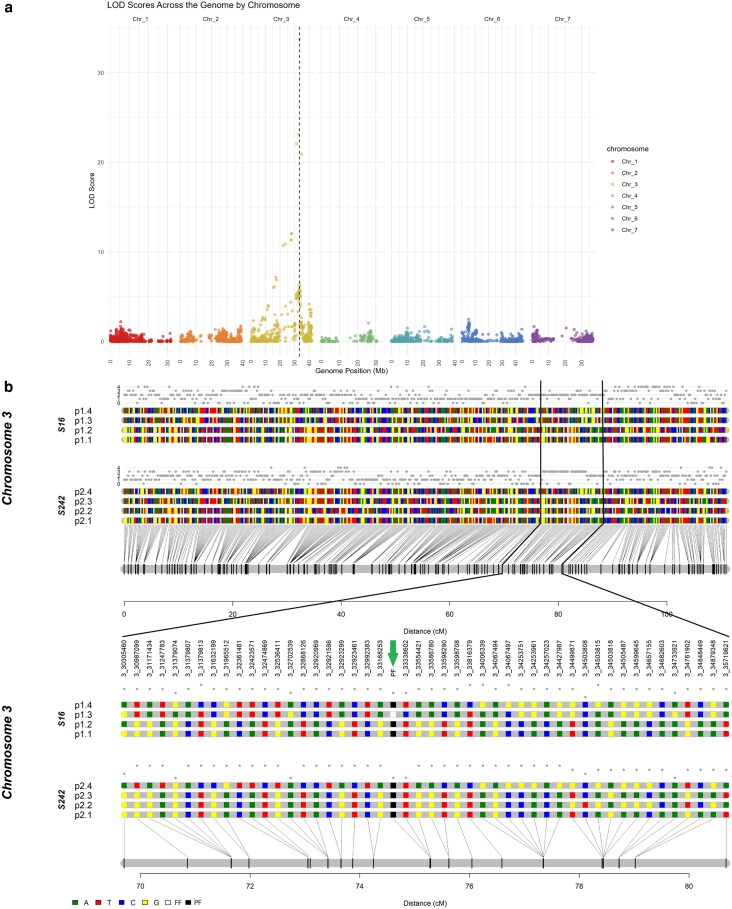
Genetic linkage analysis. (a) Recombination fraction calculation between the PF phenotype in the biparental population (presence or absence of the PF trait) and each molecular marker in the biparental population “1937”. The dashed line indicates the position of the maximum LOD score found on the chromosome 3 (Ra03). (b) Genetic linkage analysis of LG3 (upper panel) with a zoom in the region between 70 and 80 cM showing the haplotype for the SNPs linked to the marker PF2 (Ra03:33,338,602) and to the PF locus. The colored rectangles indicate SNP nucleotides within each of the four haplotypes for S16 (p1.1 to p1.4) and for S242 (p2.1 to p2.4). White and black rectangles indicate the FF and PF phenotypic allele (indicated with a downward facing arrow). The allele dosage is shown above each parent’s homologs.

The presence or absence of the PF phenotype in the biparental population was included as a phenotypic marker in the linkage map and positioned at 75.3 cM on linkage group 3 (LG3) (Fig. 4). The PF-linked marker Ra03:33,338,602 correctly classified all progeny exhibiting the PF trait as quadruplex (‘TTTT’), except for 4 individuals (1937–79, 1937–90, 1937–129, and 1937–141), which were predicted to be triplex (‘TTTC’). This marker was also positioned at 75.3 cM on LG3. The recombination fraction between each molecular marker and the PF phenotype analysis revealed a maximum LOD score of 33.7 for the marker Ra03:33,338,602. This was followed by the markers Ra03:32,702,539, Ra03:31,379,074 and Ra03:34,733,921, which had LOD scores of 23.1, 22.1, and 20.9, respectively (see Fig. 4a and Supplementary Table 4). No recombinants were identified in our mapping populations to further define the PF interval. Still, these results, combined with the GWAS analysis, suggest that the PF trait is controlled by a major recessive locus between 31 and 35 Mb on chromosome Ra03.

### Updated annotation of *R. argutus* cv. “Hillquist”

Before structural annotation, 126 Mb (42.3%) of the “Hillquist” genome was repeat-masked. The final set of predicted genes contained 30,026 coding genes, corresponding to 43,850 coding transcripts when alternative isoforms were included (Table 2). The 30,026 coding genes (calculated from the primary isoform of each gene) had an average length of 1,239 bp and a mean exon number of 5.04. Of these coding genes, 21,843 had no alternative isoforms, 5,068 had 2 isoforms, and 3,115 had 3 or more isoforms. In the predicted set of genes, 2,149 (92.4%) of 2,326 complete *R. argutus* genes orthologous to the eudicots_odb10 BUSCO families were identified, along with 76 (3.3%) genes with partial matches. A small fraction of the BUSCO families (4.3%) was not identified among the predicted *R. argutus* genes in the updated annotation (Table 2). These results suggest that the *R. argutus* cv.“Hillquist” v.1.2 assembly and annotation are 95.7% complete. Evidence supporting the predicted gene models was strong. For 79.4% of loci, the coding regions of the primary transcripts were supported by Iso-Seq or RNA-seq alignments covering at least 80% of the coding length and showing ≥80% sequence identity. Additionally, 27,328 transcripts displayed >50% peptide homology to known proteins in other plant species.

**Table 2.** Comparison of the original and updated *R. argutus* “Hillquist” genome annotations.

		v.1 (Brůna et al. 2023)	v.1.2
Gene prediction summary	Primary transcripts (loci)	38,503	30,026
	Genes with more than 1 transcript isoform	1,667	8,183
	Mean CDS length (bp)^a^	1,021	1,239
	Median coding exon length (bp)^a^	132	135
	Mean count of coding exons per gene^a^	4.36	5.04
BUSCO completedness (eudicots_odb10, *n* = 2,326)	Complete (C)	91.70%	92.40%
	Fragmented (F)	3.20%	3.30%
	Missing (M)	5.10%	4.30%
Functional annotation	Genes with PFAM domain (% of all genes)/unique PFAM domains	24,859 (65.6%)/4,324	23,976 (79.9%)/4,359
	Genes with PANTHER domain (% of all genes)/unique PANTHER domains	28,497 (74.0%)/6,243	27,517 (91.6%)/6,318
	Genes with KEGG pathway (% of all genes)/unique KEGG pathways	7,468 (19.4%)/3,290	7,699 (25.6%)/3,330
	Genes with KOG classification (% of all genes)/unique KOG domains	9,188 (23.9%)/2,467	10,186 (33.9%)/2,563
	Genes with InterPro assignment (% of all genes)/unique InterPro assignments	27,184 (70.6%)/6,353	25,517 (85.0%)/6,420
	Genes with any functional annotation (% of all genes)	31,847 (82.7%)	28,881 (96.2%)
Support for predicted gene models	PSAURON score^a^	87.9	95.4
	Genes supported by transcript alignments^a^	23,417	23,840
	% of genes supported by transcript alignments^a^	60.80%	79.40%

^a^Calculated from the primary isoform of each gene.

Functional annotation was similarly comprehensive: 28,881 (96.2%) of predicted genes received at least 1 functional annotation. Pfam domains were detected in 23,976 genes (79.9%), spanning 4,359 unique Pfam families, while PANTHER domains were identified in 27,517 genes (91.6%) across 6,318 unique families. Kyoto Encyclopedia of Genes and Genomes (KEGG) pathway mappings were assigned to 7,699 genes (25.6%), covering 3,330 unique pathways, and Eukaryotic Orthologous Group (KOG) classifications were assigned to 10,186 genes (33.9%), encompassing 2,563 unique KOG domains. Collectively, these results indicate that the majority of predicted genes in the “Hillquist” v1.2 annotation have putative biological functions supported by conserved domain, pathway, or orthology evidence.

### Putative candidate genes associated with the PF trait

Both GWAS and genetic linkage mapping identified a region between 31 and 35 Mb on chromosome Ra03 with peak significance at 33,338,602 bp as associated with the PF trait. Within this interval, 10 genes with predicted functions related to flowering regulation were identified in the “Hillquist” genome (Table 3). These include UPSTREAM OF FLC (Ruarg.3G323500), BEL1-like homeodomain 8 (*BLH8*; Ruarg.3G329500), Homeobox-leucine zipper protein REVOLUTA (*REV*; Ruarg.3G332300), and a CCCH-type zinc finger protein (Ruarg.3G334700). Additional genes in the interval include a DNA-binding protein with MIZ/SP-RING zinc finger (*SIZ1*; Ruarg.3G337900), AGAMOUS-like MADS-box protein AGL28 (Ruarg.3G338800), an AP2/ERF family transcription factor (Ruarg.3G343800), a ubiquitin-specific protease (*UBP*; Ruarg.3G346400), an AP2/B3-like transcription factor (Ruarg.3G349500), and a B3 domain-containing transcription factor *VRN1* (Ruarg.3G349600).

**Table 3.** Candidate genes for the primocane-fruiting (PF) trait in blackberry located on chromosome Ra03 between 31 and 35 mb.

Gene ID	Start position	End position	Distance from GWAS peak (Kb)	Predicted gene function	Arabidopsis homolog
Ruarg.3G323500	32004799	32009449	1329	UPSTREAM OF FLC protein	AT2G28150
Ruarg.3G329500	32620726	32627768	710	BEL1-like homeodomain 8	AT2G27990
Ruarg.3G332300	32985451	32993007	346	Homeobox-leucine zipper protein REVOLUTA	AT5G60690
Ruarg.3G334700	33264790	33269811	68	Zinc finger (CCCH-type) family protein	AT2G28450
Ruarg.3G337900	33596729	33608051	258	DNA-binding protein with MIZ/SP-RING zinc finger, PHD-finger and SAP domain-containing protein	AT5G60410
Ruarg.3G338800	33668587	33669717	330	Agamous-like MADS-box protein AGL28	AT1G01530
Ruarg.3G343800	34253342	34257478	915	Ethylene-responsive transcription factor RAP2-7 (Related to AP2.7)	AT2G28550
Ruarg.3G346400	34566835	34572407	1228	Ubiquitin-specific protease domain protein	AT5G06600
Ruarg.3G349500	34872157	34874071	1533	AP2/B3-like transcriptional factor family protein	AT1G49475
Ruarg.3G349600	34879197	34881066	1540	B3 domain-containing transcription factor VRN1	AT3G18990

### Genetic variants within the genomic region associated with the PF trait

The whole-genome resequencing data from 17 diverse tetraploid blackberry genotypes were analyzed to identify SNPs and small InDels in the genomic region on chromosome Ra03 associated with the PF trait, with the aim of pinpointing the causal variant. Genetic variants within and adjacent to potential candidate genes associated with the PF trait were first investigated. Additional variants located within 1 Mb downstream and upstream from the most significant SNP associated with the PF trait (Ra03:33,338,602) were also investigated. A total of 151,854 variants were initially detected after quality filtering in this genomic window. Because previous genetic studies (Lopez-Medina et al. 2000; Castro et al. 2013) and our GWAS results support a single recessive allele model for the PF trait, we prioritized variants where PF genotypes were homozygous for the reference allele, while FF genotypes were heterozygous or homozygous for the alternative allele. A total of 607 SNPs and small InDels were identified that fit this criterion. Of these variants, 24 were located within or adjacent to 4 of the 10 candidate genes in the region with putative functions related to flowering (Table 4). One small InDel located at 32,625,674 bp was identified in an intron of the gene Ruarg.3G329500, which encodes for a BEL1-like homeodomain 8 (*BLH8*). Two SNPs and 1 InDel variant were identified in the intron region and 1 InDel within 1 Kb upstream of the gene Ruarg.3G332300, which codes for a Homeobox-leucine zipper protein REVOLUTA. Twelve variants were identified within and adjacent to the CCCH-type Zinc finger family protein-coding gene (Ruarg.3G334700), including 3 intron variants, 1 synonymous SNP in an exon region, two 3′UTR variants, and 6 variants within 1 Kb downstream of the gene. Furthermore, 7 variants were found within or adjacent to the ubiquitin-specific protease (Ruarg.3G346400), including 2 intron variants, 3 SNPs in the 3′UTR region and 2 SNP within 1 Kb downstream of the gene.

**Table 4.** Variants identified within and adjacent to potential candidate genes in the primocane-fruiting (PF) locus in blackberry, with functional annotations related to flowering development, based on whole-genome sequencing data from 17 blackberry selections and cultivars.

Chrom	Position	Reference allele	Alternative allele	Annotation	Gene ID
Ra03	32625674	CATATAT	CATATATATAT,C,CATATATATATATAT	intron variant	Ruarg.3G329500
Ra03	32987425	G	A	intron variant	Ruarg.3G332300
Ra03	32990834	G	GA	intron variant	Ruarg.3G332300
Ra03	32991091	G	A,*,C	intron variant	Ruarg.3G332300
Ra03	32993429	CAA	CA,CAAA,C,*	upstream variant	Ruarg.3G332300
Ra03	33264754	TCGTACA	T	downstream variant	Ruarg.3G334700
Ra03	33264755	C	*,T	downstream variant	Ruarg.3G334700
Ra03	33264759	C	*,T	downstream variant	Ruarg.3G334700
Ra03	33264762	A	G	downstream variant	Ruarg.3G334700
Ra03	33264772	C	T	downstream variant	Ruarg.3G334700
Ra03	33264775	C	T	downstream variant	Ruarg.3G334700
Ra03	33264798	A	T	3′ UTR variant	Ruarg.3G334700
Ra03	33264800	T	TTC	3′ UTR variant	Ruarg.3G334700
Ra03	33266514	AAG	A,*	intron variant	Ruarg.3G334700
Ra03	33266577	AAAGGGCATACTATATCTTTTTTTGAATAG	A	intron variant	Ruarg.3G334700
Ra03	33267506	C	T	synonymous variant	Ruarg.3G334700
Ra03	33268358	C	T	intron variant	Ruarg.3G334700
Ra03	34566811	G	T	downstream variant	Ruarg.3G346400
Ra03	34566832	A	C	downstream variant	Ruarg.3G346400
Ra03	34566857	A	G	3′ UTR variant	Ruarg.3G346400
Ra03	34566957	A	C	3′ UTR variant	Ruarg.3G346400
Ra03	34566987	C	T	3′ UTR variant	Ruarg.3G346400
Ra03	34567383	AAT	A,ACAT,*	intron variant	Ruarg.3G346400
Ra03	34569819	A	C	intron variant	Ruarg.3G346400

These variants distinguish PF (6 genotypes) from FF (11 genotypes) based on the WGS data. Asterisk represents a deletion spanning over the variant position.

The additional 583 genetic variants detected within the 2 Mb window around the GWAS peak were all located in either intergenic regions or within noncandidate genes (Supplementary Table 5). From this set of variants, 12 SNP markers were selected for KASP development; the 2 most strongly associated with the PF trait in the GWAS located at 33,338,602 and 33,338,650 bp, and 10 other SNPs that discriminate between the PF and FF genotypes at positions 33,713,953 bp, 33,968,573 bp, 34,098,961 bp, 34,100,366 bp, 34,100,510 bp, 34,106,515 bp, 34,287,111 bp, 34,352,079 bp, 34,364,423 bp and 34,399,952 bp (Supplementary Tables 6 and 7).

### Development and validation of diagnostic KASP markers

**Fig. 5.**
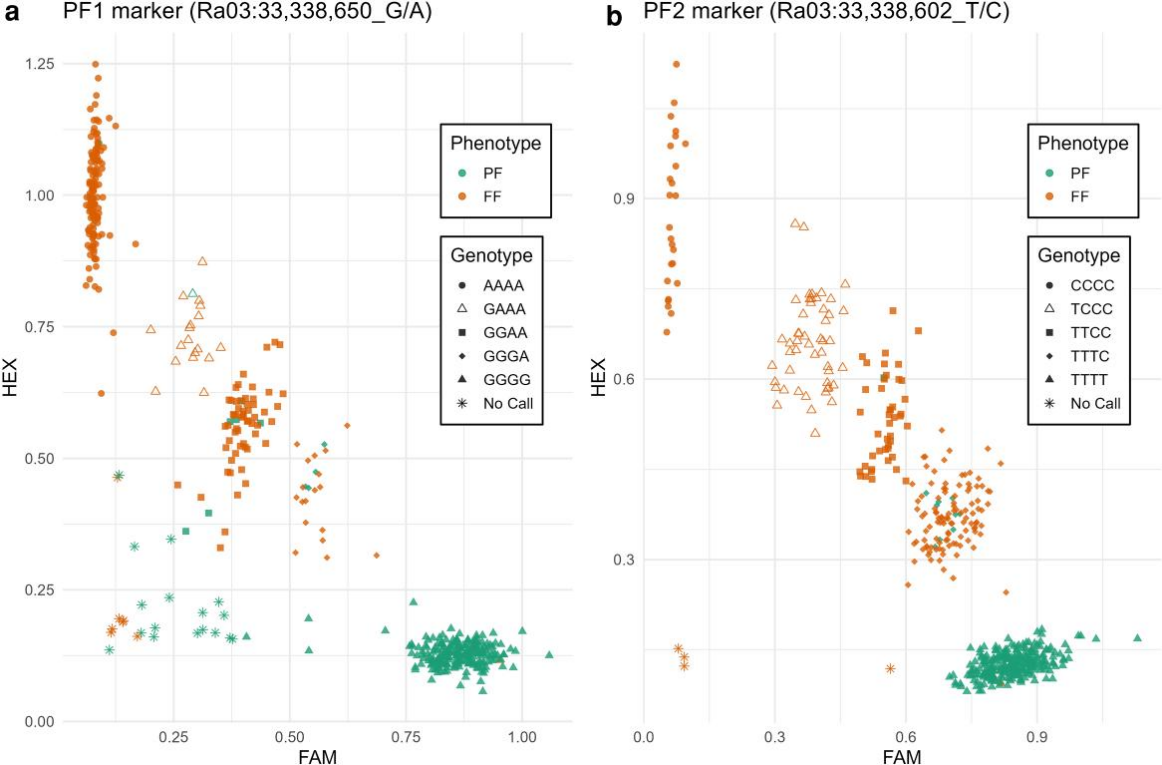
Validation of KASP markers. The scatter plot displays the FAM and HEX values for 647 genotypes for marker *PF1* a), which targets the SNP at Ra03: 33,338,650 (G/A), and marker *PF2* b), which targets the SNP at Ra03: 33,338,602 (T/C). The allele dosage for the simplex, duplex, and triplex classes was determined based on the fluorescence intensity of the FAM and HEX dyes.

Among the 12 KASP markers, 2 were identified as the most predictive in the validation panel. These 2 markers, targeting the SNPs located at 33,338,650 and 33,338,602 bp on chromosome Ra03, named *PF1* and *PF2*, respectively, predicted the PF phenotype with 96.8 and 96.5% accuracy, in the validation panel (Fig. 5 and Supplementary Table 8). These SNPs were the markers most strongly associated with the PF trait in the GWAS analysis (Fig. 2 and Supplementary Table 2).

The *PF1* and *PF2* marker reactions failed to call alleles for 24 and 4 of 494 genotypes in the validation panel, respectively. Among the genotypes with successful allele calls, 455 and 473 were correctly predicted by *PF1* and *PF2* markers, respectively. Both markers incorrectly predicted 11 genotypes. Five seedlings from population 1937 (1937–001, 1937–079, 1937–090, 1937–129, and 1937–141), 3 selections from the UADA breeding program (S307, S441, and S443), and 2 selections from Hortifrut Genetics Ltd. (HFG39 and HFG45) were all scored as PF genotypes but had at least 1 nonPF allele at both *PF1* and *PF2* sites (Supplementary Table 8). On the other hand, 1 seedling from population 1937 (1937–029) was scored as a FF genotype but was predicted to be homozygous for the PF allele at both *PF1* and *PF2* markers.

Six additional genotypes were inaccurately predicted by only 1 of the 2 markers. One selection from the USDA-ARS HCPGIR germplasm (OR72) and one from the UADA breeding program (S306) were scored as PF genotypes but were predicted to have only 2 PF alleles for the *PF1* marker. Finally, 1 seedling from population 1937 (1937–107), 1 selection from the UADA program (S140), and 2 selections from the USDA-ARS HCPGIR germplasm (OR34 and OR53) were scored as FF genotypes but were predicted to have only PF alleles for the *PF2* marker.

## Discussion

### A single major locus on chromosome Ra03 controls PF in tetraploid blackberries

Genome-wide association analysis across 365 tetraploid blackberry genotypes revealed a single major locus on chromosome Ra03 strongly associated with the PF trait. Using 81,064 high-quality biallelic SNPs distributed across the 7 blackberry chromosomes, 419 SNPs were significantly associated with PF within a 20.5 Mb region on Ra03 (21.2–41.7 Mb), with the most strongly associated variants located at 33,338,602 and 33,338,650 bp (−log_10_*P* = 183.8). Nearly all PF genotypes (97%) were homozygous for the reference allele at this locus, while all FF genotypes had at least 1 copy of the alternative allele. This clear segregation pattern indicates that a single, major-effect recessive locus underlies PF in tetraploid blackberry.

The GWAS findings were independently validated through linkage analysis in a biparental F_1_ population derived from a cross between the FF selection “S16” and the PF selection “S242”. The PF phenotype mapped to linkage group 3 (LG3) at 75.3 cM and was tightly linked to a KASP marker (*PF2*) targeting the peak GWAS SNP Ra03:33,338,602. The maximum LOD score for association with PF was observed at marker Ra03:33,338,602 (LOD = 33.7), followed by Ra03:32,702,539 (LOD = 23.1), Ra03:31,379,074 (LOD = 22.1) and Ra03:34,733,921 (LOD = 20.9). Taken together, the GWAS and linkage results indicate that PF in blackberry is controlled by a major recessive locus located between 31 and 35 Mb on chromosome Ra03.

Earlier work by Castro et al. (2013) placed the PF locus between SSR markers RH_MEa0006aC04-175 and RH_MEa0007aG06-152, which were initially assigned to linkage group 7 but later shown to align to chromosome Ra02 of the “Hillquist” genome assembly. No associations with the PF trait were detected on Ra02 or Ra07 in the present study, suggesting that the earlier placement may have resulted from limited marker density and the absence of a reference genome. In contrast, the current study used 3,197 SNP markers to construct a high-density linkage map spanning 769.4 cM across 7 chromosomes, providing substantially higher resolution and collinearity with the reference genome. Unlike previous studies limited to single crosses, our approach also captures the genetic diversity and allelic structure present in breeding germplasm, enabling more accurate localization of the PF locus and identification of tightly linked diagnostic SNPs suitable for marker-assisted selection.

The PF mapping results in blackberry contrast with those reported for red raspberry, where Jibran et al. (2019) identified 2 quantitative trait loci, RiAF3 and RiAF4, located on chromosomes 3 and 4 of *R. idaeus*. These regions correspond to chromosomes Ra03 (0–9 Mb) and Ra04 (31–32 Mb) of the *R. argutus* cv. Hillquist genome, neither of which overlapped the PF locus identified here. The lack of shared loci or candidate genes suggests that primocane fruiting in tetraploid blackberry and red raspberry may have evolved through distinct genetic mechanisms. Together, the GWAS and linkage mapping results firmly establish the PF locus on chromosome Ra03 as the major determinant of annual flowering in tetraploid blackberry.

### Additional insights from high-density linkage mapping in blackberry

We constructed the first integrated high-density genetic linkage map for tetraploid blackberry using 113 progeny from a cross between the FF selection “S16” and the PF selection “S242”. The final map spanned 769.4 cM and included 3,197 nonredundant SNP markers, averaging ∼4 markers per cM across 7 LGs corresponding to the basic chromosome number in blackberry. All 7 linkage groups exhibited high collinearity with the *R. argutus* cv. “Hillquist” reference genome (Supplementary Fig. 3), confirming the accuracy of marker ordering and alignment. The fewest markers and largest gap were observed on LG4 (Ra04), consistent with reduced recombination and genetic diversity in the distal region of this chromosome. This region coincides with the prickle-free locus, which has been shown to contain an extensive linkage disequilibrium block (Chizk et al. 2023; Johns et al. 2025). Because both parents of the mapping population are prickle-free, the paucity of markers on Ra04 may reflect historical selection and a resulting loss of heterozygosity in this region.

This map represents a substantial advance in marker density and genomic coverage compared with the first SSR-based blackberry map of Castro et al. (2013), which comprised only 119 markers and lacked the resolution to anchor linkage groups to specific chromosomes. It also improves upon the pseudo-testcross maternal map of the breeding selection “A-2551TN” (Brůna et al. 2023) by incorporating markers across all dosage classes and producing a fully integrated representation of all 4 homologs for each chromosome, rather than relying solely on simplex markers to generate haplotype-resolved but nonintegrated linkage groups.

No evidence of preferential pairing among homologs was detected in either parent (Supplementary Fig. 4), suggesting predominantly polysomic inheritance in tetraploid blackberry. In polyploid species, double reduction can occur when multivalent pairing causes sister chromatid segments to migrate into the same gamete during meiosis (Mather 1936; Bourke et al. 2015), thereby increasing homozygosity and altering expected segregation ratios. In this mapping population, the FF female parent “S16” had 3 copies of the PF allele (TTTC) at SNP marker Ra03:33338602 (*PF2*), while the PF parent “S242” had 4 copies of the PF allele (TTTT). A single FF progeny (1937–131) exhibited the duplex genotype (TTCC) for the *PF2* locus. Previously, Vaughn et al. (2023) reported 6 progenies with the duplex genotype in this population; however, 5 were excluded from the linkage map as nontrue seedlings in the PCA analysis, most likely resulting from pollen contamination or self-pollination (Supplementary Fig. 2). The remaining progeny, 1937-131, is most likely an actual F_1_ seedling of the “S16”×“S242” cross, providing possible evidence of low-frequency double reduction in this mapping population. Collectively, these results establish a robust, genome-aligned linkage framework for tetraploid blackberry that captures the full complexity of polysomic inheritance and provides a foundation for future trait mapping and comparative genomic analyses.

### An improved annotation of *R. argutus* cv. “Hillquist”

The updated *R. argutus “*Hillquist” annotation (v1.2) represents a substantial improvement over the original annotation, both in gene model accuracy and functional characterization. The final set of 30,026 coding genes is significantly smaller than the original 38,503, reflecting more stringent filtering of likely false predictions: the original annotation included 9,407 genes with no transcriptomic or protein homology support. Despite fewer predicted genes, the updated annotation exhibits higher completeness and confidence, with more BUSCO eudicot markers recovered, better transcriptome support (both in relative and absolute terms) and more comprehensive functional annotation. In particular, compared with the original annotation, the updated version shows consistently higher proportions of genes assigned to Pfam, PANTHER, KEGG pathways, and KOG categories, as well as a large increase in the percentage of genes receiving any functional annotation (82.7% vs 96.2%). These improvements are accompanied by increases in the number of unique domains or pathways captured across Pfam, PANTHER, KEGG, and KOG classifications, reflecting broader and more diverse functional coverage (Table 2). Together, these gains indicate that the improved structural annotation is matched by richer and more reliable biological characterization. Finally, the updated annotation achieved a markedly better Protein Sequence Assessment Using a Reference ORF Network (PSAURON) score, with PSAURON providing a genome-wide measure of protein-coding sequence accuracy using a machine learning model trained across diverse taxa (Sommer et al. 2025).

Several methodological improvements contributed to these gains. Repeat-masking was refined by manually curating predicted transposable element libraries to prevent genuine genes from being erroneously masked. The annotation pipeline also fully integrated long-read Iso-Seq data, rather than using it solely for validation of gene models and minor repeat-masking refinement in the original annotation. This integration enabled correction of mispredicted gene models and improved gene prediction in regions lacking homology to known proteins. Furthermore, the set of protein homologs used for functional annotation was curated to include additional close relatives, improving the accuracy of homology-derived models. The updated annotation also includes UTR predictions, which may be particularly relevant for functional analyses of regulatory variants, and a significantly larger set of predicted alternative isoforms. Overall, these improvements provide a more accurate and biologically meaningful representation of the *R. argutus* gene space, supporting downstream analyses including candidate gene identification and variant interpretation for traits such as PF.

### Putative candidate genes for PF

The PF locus on chromosome Ra03 was defined by both GWAS and genetic linkage mapping, with the peak association in both analyses located at 33,338,602 bp. To identify candidate genes, we examined annotated genes in the 31–35 Mb interval of Ra03 with homology to known flowering regulators. Ten genes were highlighted as candidates based on putative roles in flowering: Ruarg.3G323500 (*UFC*), Ruarg.3G329500 (*BLH8*), Ruarg.3G332300 (*REV*), Ruarg.3G334700 (CCCH-type zinc finger), Ruarg.3G337900 (*SIZ1*), Ruarg.3G338800 (*AGL28*), Ruarg.3G343800 (*RAP2-7*), Ruarg.3G346400 (*UBP*), Ruarg.3G349500 (*AP2*/*B3*-like), and Ruarg.3G349600 (*VRN1*) (Table 3).

Among the 10 candidate genes in the locus, 4 harbor genetic variants that segregate with the PF phenotype (Table 4). Ruarg.3G329500, a homolog of the *BEL1*-like homeodomain 8 (*BLH8*, also called *POUND-FOOLISH*) plant homeobox transcription factor coding gene, contains a single intronic InDel (32,625,674 bp) distinguishing PF and FF genotypes. Functional annotation indicated that this and all other PF-associated intronic variants identified in candidate genes were not predicted to disrupt canonical splice donor or acceptor motifs or conserved splice-region elements required for intron processing (Supplementary Table 5). In *Arabidopsis*, *BLH8*, works together with another *BELL1*-like homeodomain protein, *PENNYWISE* (*PNY*), to maintain the shoot apical meristem (SAM) and regulate floral transition (Ung et al. 2011). Another homeobox TF found in this region, Ruarg.3G332300, also harbors 3 intronic SNP variants and 1 InDel within 1 Kb upstream of the gene that segregate with PF. Ruarg.3G332300 is a homolog of the Class III Homeodomain-leucine zipper protein *REVOLUTA* (*REV*), which is necessary for shoot apical meristem development (Talbert et al. 1995) and required for the initiation of both lateral shoot meristems and flower meristems (Otsuga et al. 2001).

One homolog of a ubiquitin-specific protease, Ruarg.3G346400, was also found in the genomic region associated with the PF trait. *UBP* has been implicated in flowering time regulation through the photoperiod pathway. Mutations in *UBP12* and *UBP13* promote earlier expression of *CONSTANS*, which leads to increased expression of *FT* and earlier flowering in *Arabidopsis* (Cui et al. 2013). Ruarg.3G346400, contains 2 intronic variants, 3 SNP variants in the 3′UTR and 2 SNP variants within 1 Kb downstream of the gene differentiating PF and FF genotypes (Table 4). While these changes may have functional consequences, Ruarg.3G346400 is located over 1.2 Mb from the GWAS peak, making it a less likely causal gene. Moreover, UBP acts as a flowering promoter; a loss-of-function mutation would be expected to delay flowering rather than confer the recessive PF phenotype. This contrasts with the expected behavior of a mutation in a flowering repressor, consistent with the recessive inheritance of primocane fruiting.

By contrast, Ruarg.3G334700, a CCCH-type zinc finger gene, is positioned just 68 Kb from the GWAS peak and harbors the highest density of PF-associated variants within the locus: 3 intronic variants, two 3′ UTR variants, 6 variants within 1 kb downstream, and 1 synonymous exonic SNP. Members of the CCCH zinc finger family have been implicated in flowering regulation across multiple species. In *Arabidopsis*, *KHZ1* and *KHZ2* promote floral transition, with double mutants displaying delayed flowering and overexpression leading to early flowering (Yan et al. 2017). Similar flowering-promoting roles have been observed for *AaZFP3* in *Adonis amurensis* and CpC3H3 in *Chimonanthus praecox* (Liu et al. 2022; Wang et al. 2022), whereas overexpression of the alfalfa *MsZFN* gene led to delayed flowering (Chao et al. 2014), highlighting that CCCH proteins can act as either promoters or repressors depending on context. Importantly, FF blackberries are short-day plants, while most functional data on these homologs come from long-day species, which may alter regulatory networks controlling flowering. The close proximity of Ruarg.3G334700 to the GWAS peak, coupled with the concentration of PF-associated variants, makes it an interesting candidate for the PF phenotype in blackberry.

The remaining 6 genes in the 31–35 Mb interval [Ruarg.3G323500 (*UFC*), Ruarg.3G337900 (*SIZ1*), Ruarg.3G338800 (*AGL28*), Ruarg.3G343800 (*RAP2-7*), Ruarg.3G349500 (*AP2/B3*-like), and Ruarg.3G349600 (*VRN1*)] have annotated roles consistent with flowering regulation. *UFC* is located upstream of *FLC*, a major flowering repressor in *Arabidopsis*, although *UFC*'s direct role in flowering remains unclear, and neither of the publicly available blackberry genomes harbors a clear *FLC* homolog (Finnegan et al. 2004; Aljaser et al. 2022). *SIZ1* acts as a floral repressor via SUMOylation of flowering regulators (Jin et al. 2008; Miura et al. 2013; Son et al. 2014). *AGL28* and *RAP2-7* are involved in promoting floral meristem identity (Yoo et al. 2006; Zhang et al. 2015; Agarwal and Khurana 2019; Du et al. 2020; Li et al. 2025), and *AP2/B3*-like transcription factors can modulate the photoperiodic pathway, promoting or repressing flowering through direct binding to the promoters of *SOC1* and *FT* (Hu et al. 2021; Yu et al. 2020). Despite their plausible functions, none of these genes harbor variants that segregate with the PF trait, suggesting they are unlikely to be the primary causal gene in this population.

### Diagnostic KASP markers for PF

The KASP markers developed in this study, *PF1* and *PF2*, demonstrated strong predictive ability for the PF phenotype, correctly classifying 96.8 and 96.5% of genotypes, respectively, across a large and diverse validation panel. These are among the first diagnostic molecular markers developed for a major agronomic trait in blackberry. Previously, the only other diagnostic markers linked to a trait of economic importance were those associated with the prickle-free cane phenotype (Johns et al. 2025).

The development of reliable diagnostic markers for PF represents a major advance for blackberry breeding. Although PF cultivars offer significant production advantages, including extended harvest seasons, the ability to schedule fruiting through primocane management, avoidance of winter injury, and adaptation to low-chill environments, they generally lag behind elite FF cultivars in fruit quality, firmness, and flavor (Clark 2008). Improving PF germplasm therefore requires crosses between high-quality FF selections and PF parents. Because PF is recessively inherited in a multisomic tetraploid background, only 1 in 6 seedlings (≈17%) from a duplex FF (CCTT) × PF (TTTT) cross is expected to express the PF phenotype. This low-frequency greatly increases the population size, space, and time required to recover PF progeny with elite fruit quality. Reliable markers enable breeders to identify FF parents that carry PF alleles and to cull FF seedlings prior to planting, thereby enriching the proportion of PF individuals in segregating populations and accelerating the development of high-quality PF cultivars.

The PF1 and PF2 markers were broadly effective across germplasm from multiple breeding programs, correctly predicting the phenotype of 37 of 39 selections from Hortifrut Genetics Ltd., 73 of 76 selections from the USDA-ARS HCPGIR program, and all accessions from the USDA-ARS-NCGR collection. This demonstrates that the markers are robust and transferable beyond the UADA breeding program, a critical feature for adoption in both public and private breeding efforts.

Most inconsistencies between phenotypic and genotypic predictions were observed in population 1937. This is likely due to challenges in scoring PF among seedlings, which were planted at high-density, where primocanes from neighboring plants can intermingle. Additionally, basal flowering can sometimes be mistaken for true primocane fruiting when scoring individual plants. Such phenotyping ambiguity likely explains a substantial portion of the observed discrepancies between marker prediction and field phenotype. Three UADA selections (S307, S441, and S443) were scored as PF but were predicted to carry at least 1 FF allele at both *PF1* and *PF2* loci. All 3 share the same FF parent (S34), which displays strong basal flowering on floricanes late in the season when primocanes are actively flowering. These half-sib selections consistently exhibited <30% of primocanes flowering in any given year, suggesting that their ambiguous phenotype may result from alleles at a secondary locus influencing basal or partial primocane flowering, rather than recombination at the primary PF locus.

Both *PF1* and *PF2* markers target SNPs within an intron of Ruarg.3G335600, a homolog of β-galactosidase 8 (BGAL8). As this gene has no known role in flowering, the associated variants are unlikely to be causal. This raises the possibility that a small number of true recombinants exist in the population, reflecting historical crossover events within the linkage disequilibrium block surrounding the PF locus. Overall, the *PF1* and *PF2* markers performed exceptionally well across diverse germplasm and provide a powerful, easy-to-use tool for accelerating selection in blackberry breeding programs. These markers are already being used to identify PF allele carriers among elite FF parents, plan crosses, and cull nonPF seedlings before planting in the UADA breeding program. Broader implementation of these diagnostic markers will enhance breeding efficiency across programs and facilitate the continued improvement of fruit quality and yield in primocane-fruiting blackberry cultivars.

## Conclusions

This study provides strong evidence that a single recessive locus between 31 and 35 Mb on chromosome Ra03 controls the PF trait in blackberry. The identification of this locus through both GWAS and linkage mapping represents a major advance in understanding the genetic basis of PF, a trait of exceptional economic importance in blackberry and raspberry whose genetic control has remained largely unresolved. Within this region, several candidate genes with annotated roles in flowering regulation were identified, and allele mining using whole-genome resequencing data revealed numerous variants distinguishing PF and FF genotypes. Notably, variants in the 3′ untranslated region of a CCCH-type zinc finger gene, located ∼68 kb from the GWAS peak, emerged as a particularly promising candidate for further study. The availability of a high-quality, chromosome-scale genome annotation enabled detailed investigation of gene content and allelic variation within the PF locus, laying the groundwork for future functional analyses. Gene expression profiling of shoot apical meristems and targeted functional validation will be critical to determine causal variants for PF. Finally, the development of 2 highly accurate KASP markers (*PF1* and *PF2*) tightly linked to the PF locus provides practical tools for marker-assisted selection, enabling more efficient development of high-quality PF cultivars. Together, these results advance our understanding of flowering regulation in short-day Rosaceae species and provide key resources for both fundamental and applied blackberry genetics.

## Supplementary Material

iyag078_Supplementary_Data

## Data Availability

The updated genome annotation for *R. argutus* cv. “Hillquist” v.1.2 is available on Phytozome (https://phytozome-next.jgi.doe.gov/info/Rargutus_v1_2) and can also be accessed at the Genome Database for Rosaceae (tfGDR1090; https://www.rosaceae.org/publication_datasets). Raw Isoseq and Illumina transcriptome data for *R. argutus* cv. Hillquist are available at NCBI under Bioproject IDs PRJNA830911and PRJEB36280 (BioSamples SAMEA6502409, SAMEA6502410, SAMEA6502411, SAMEA6502412, and SAMEA6502413), respectively. The whole-genome resequencing data used in this study are also publicly available at NCBI under Bioproject PRJNA1002337. The Capture-Seq derived SNP data used in GWAS are available in the Genome Database for Rosaceae repository (accession number tfGDR1069). All other data used in this study are presented in this manuscript and its Supplementary files.
