# Supplementary material for: A single genomic region controls primocane fruiting in tetraploid blackberry
Source: Genetics. 2026 Mar 23;233(2):iyag078. doi: 10.1093/genetics/iyag078 (PMC13232755; doi:10.1093/genetics/iyag078)
Supplement: iyag078_Supplementary_Data [file iyag078_supplementary_data.zip › Supplemental_File_1_GENETICS-2025-308894.docx]

## Supplementary Table Legends:

Supplementary Table 1. Blackberry selections and cultivars included in the genome-wide association analysis, their phenotypic description as Primocane-Fruiting (PF) or Floricane-Fruiting (FF) genotypes, and the allele dosage for the highest associated SNP markers at 33,338,602 and 33,338,650 bp on chromosome Ra03.

Supplementary Table 2. A total of 419 significant SNPs associated with the primocane-fruiting trait in the genome-wide association with -log10(p) values and effects for the simplex dominance (1-dom-ref) model.

Supplementary Table 3. Genotypic data for the biparental population ‘1937’ (113 progeny) derived from a cross between the FF genotype ‘S16’ and the PF genotype ‘S242’, along with their phenotypic classification as PF (Primocane-Fruiting) or FF (Floricane-Fruiting).

Supplementary Table 4. 53 SNPs highly linked with the Primocane-Fruiting phenotype in the blackberry biparental population '1937'. LOD score of the recombination fraction between each molecular marker and the PF phenotype was calculated, and a maximum LOD score was observed for the marker 3_33338602 .

Supplementary Table 5. List of genetic variants located within 1 Mb upstream and downstream of the GWAS peak associated with the primocane‑fruiting trait on chromosome Ra03 differentiating Primocane-Fruiting (PF) and Floricane-Fruiting (FF) genotypes.

Supplementary Table 6. Allele dosage of 17 blackberry selections and cultivars, sequenced by whole-genome sequencing, for 12 SNP targets selected for KASP assay design to discriminate between PF and FF genotypes.

Supplementary Table 7. Primer sequence for Kompetitive Allele Specific Primer (KASP) assays targeting SNP distributed trough the genomic region associated with the PF trait on chromosome Ra03.

Supplementary Table 8. KASP markers validation panel. Genotypes used in the KASP validation study, phenotype for the Primocane-Fruiting (PF) trait (presence/absence), germplasm source, and information for the two most predictive KASP markers (PF1, Ra03:33338650 and PF2, Ra03:33,338,602), including FAM and HEX fluorescence value and allele dosage.

## Supplementary Figure Legends:

Supplementary Figure 1. Chromosome-specific QQ-plots for the association analysis between the Primocane-Fruiting trait and SNPs under the simplex-dominant (1-dom) model.

Supplementary Figure 2. PCA for the biparental population ‘1937’ derived from the cross between the FF genotype ‘S16’ and the PF genotype ‘S242’. Samples highlighted in red were excluded from the genetic linkage analysis.

Supplementary Figure 3. Comparison of the blackberry tetraploid genetic linkage map with the ‘Hillquist’ blackberry (*R. argutus*) physical map. As expected, seven linkage groups were identified for chromosomes Ra01 to Ra07. No noticeable inversions or translocations were detected.

Supplementary Figure 4. Preferential pairing profile in a tetraploid blackberry biparental population. (A) Probability profiles for homolog pairs in the parents ‘S16’ (P1) and ‘S242’ (P2) across 7 LGs. The dashed lines specify the pairing probability expected under random pairing. (B) *-log10(P)* of a χ 2 independence test for all possible homolog pairs where dashed lines indicate *P* < ${10}^{-2}$. No significant level of preferential pairing was observed across S16 or S242 parent.
