# Supplementary figures and images for: A single genomic region controls primocane fruiting in tetraploid blackberry
Source: Genetics. 2026 Mar 23;233(2):iyag078. doi: 10.1093/genetics/iyag078 (PMC13232755; doi:10.1093/genetics/iyag078)

# QQ-plot

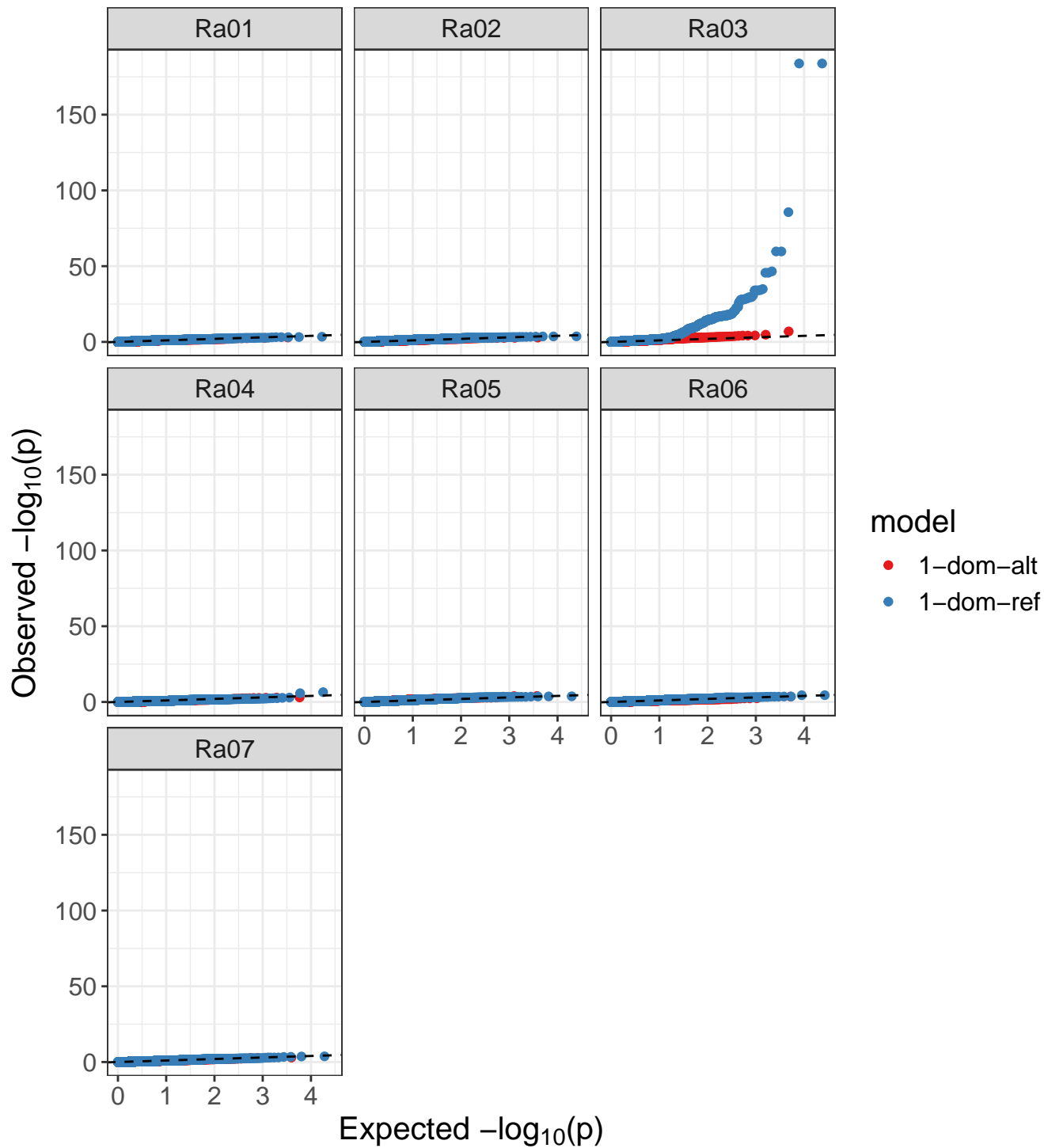

Supplement: iyag078_Supplementary_Data [file iyag078_supplementary_data.zip › Supplemental_Figure_1_GENETICS-2025-308894.pdf]

# Individuals - PCA

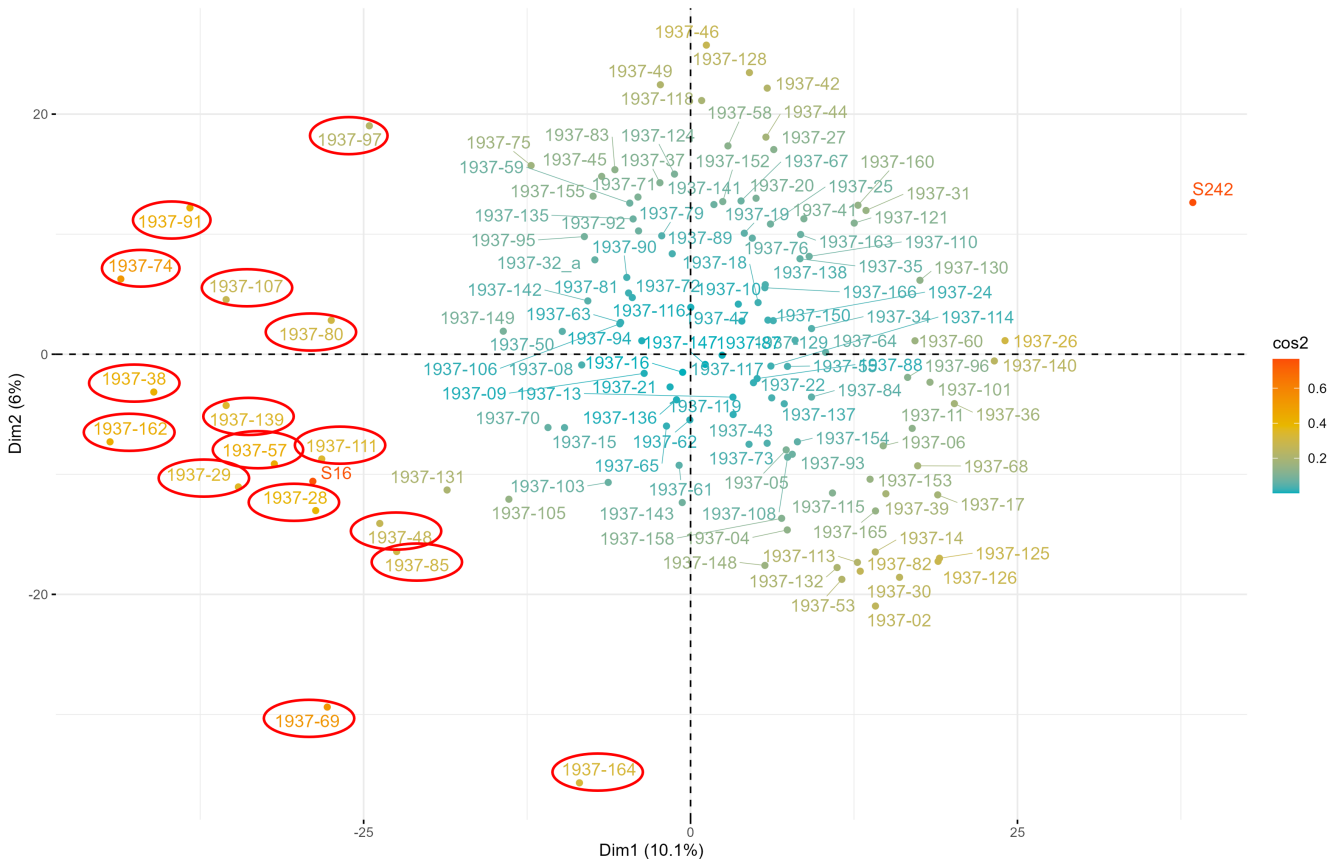

Supplement: iyag078_Supplementary_Data [file iyag078_supplementary_data.zip › Supplemental_Figure_2_GENETICS-2025-308894.pdf]

Linkage group

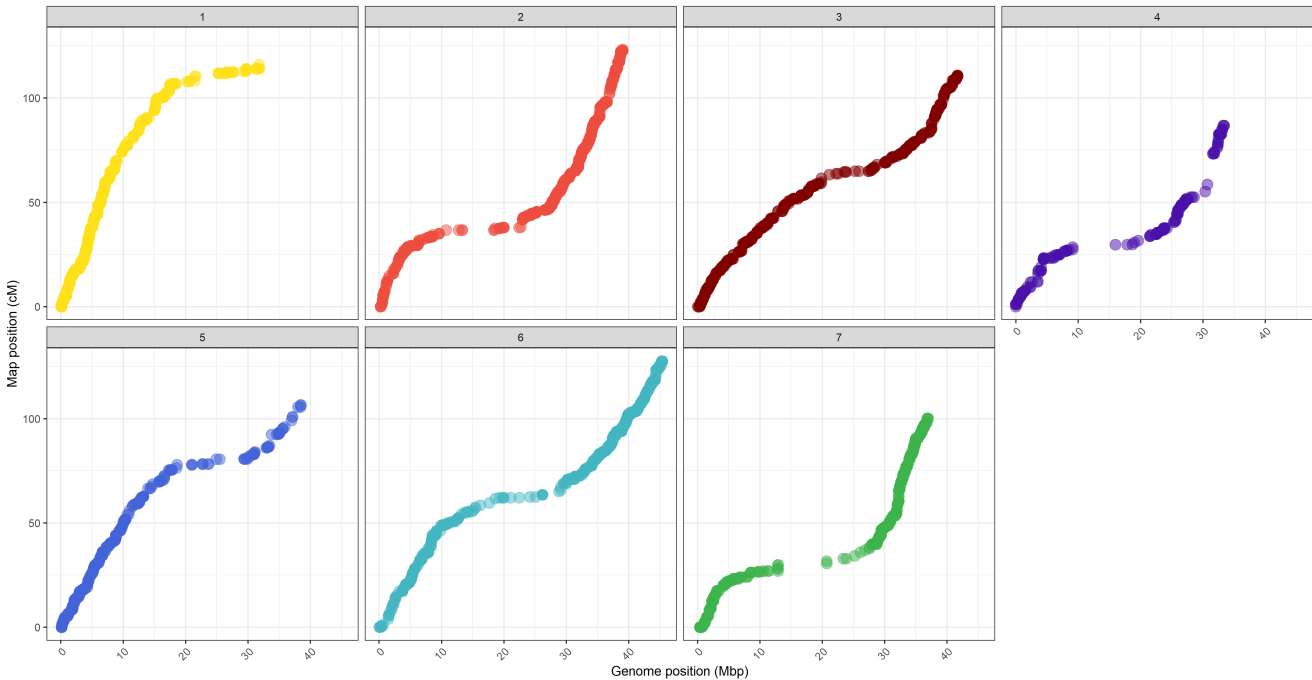

Supplement: iyag078_Supplementary_Data [file iyag078_supplementary_data.zip › Supplemental_Figure_3_GENETICS-2025-308894.pdf]

Linkage group

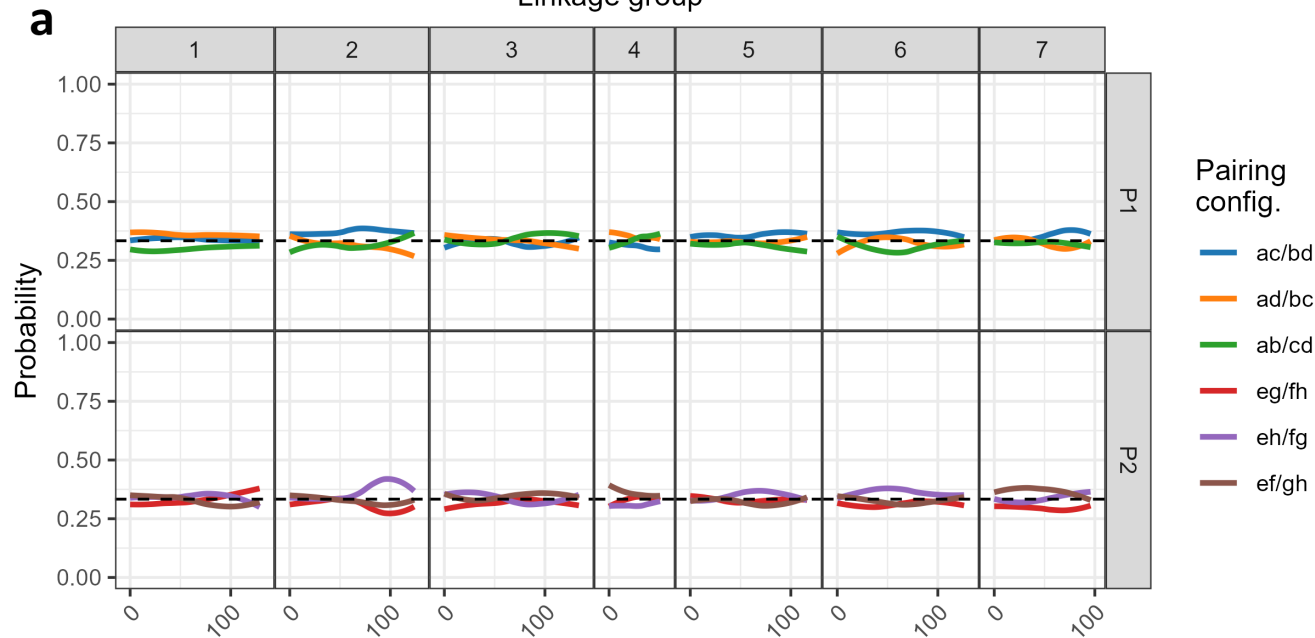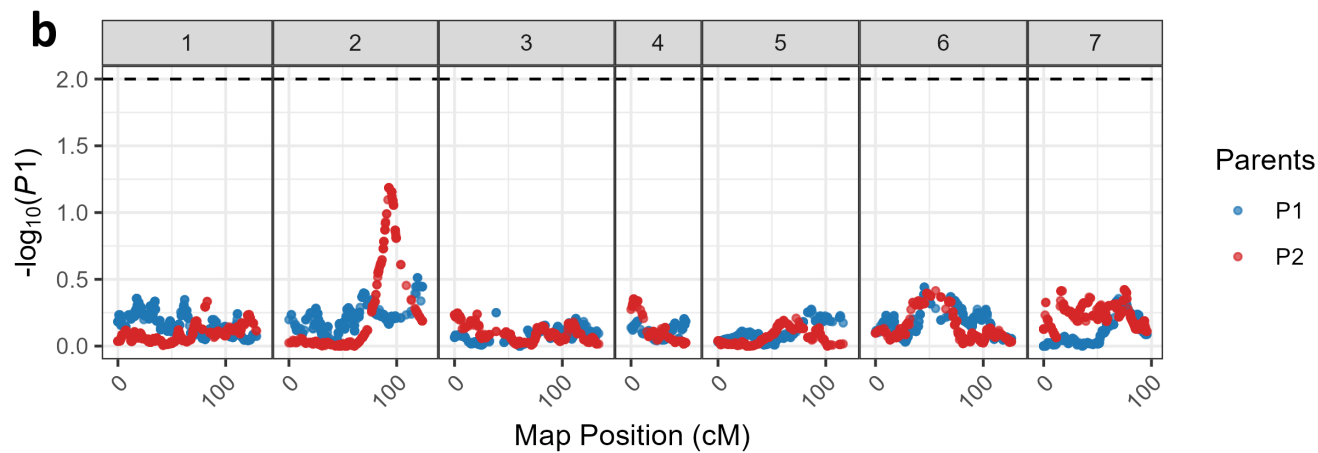

Supplement: iyag078_Supplementary_Data [file iyag078_supplementary_data.zip › Supplemental_Figure_4_GENETICS-2025-308894.pdf]
